# Simultaneous detection of bovine viral diarrhea virus (BVDV) and bovine herpesvirus 1 (BoHV-1) using recombinase polymerase amplification

**DOI:** 10.1038/s41598-024-56869-7

**Published:** 2024-05-03

**Authors:** Lingling Jiang, Gang Zhang, Pu Wang, Xiaoxia Niu, Qiang Liu, Sinong Zhang, Weifeng Gao, Yong Li

**Affiliations:** 1 School of Life Sciences, Ningxia University, Yinchuan, China; 2 Key Laboratory of Ministry of Education for Conservation and Utilization of Special Biological Resources in Western China, Ningxia University, Yinchuan, China

**Keywords:** Biochemistry, Biological techniques, Molecular biology, Zoology

## Abstract

Bovine viral diarrhea virus (BVDV) is considered to be the most common agent of severe diarrhea in cattle worldwide, causing fever, diarrhea, ulcers, and abortion. Bovine herpesvirus 1 (BoHV-1) is also a major bovine respiratory disease agent that spreads worldwide and causes extensive damage to the livestock industry. Recombinase polymerase amplification (RPA) is a novel nucleic acid amplification method with the advantages of high efficiency, rapidity and sensitivity, which has been widely used in the diagnosis of infectious diseases. A dual RPA assay was developed for the simultaneous detection of BVDV and BoHV-1. The assay was completed at a constant temperature of 37 °C for 30 min. It was highly sensitive and had no cross-reactivity with other common bovine viruses. The detection rate of BVDV RPA in clinical samples (36.67%) was higher than that of PCR (33.33%), the detection rate of BoHV-1 RPA and PCR were equal. Therefore, the established dual RPA assay for BVDV and BoHV-1 could be a potential candidate for use as an immediate diagnostic.

## Introduction

Bovine viral diarrhoea viruses (BVDV), with positive, non-segmented, single stranded RNA genome, comprise a heterogeneous group of viruses which belong to the genus *Pestivirus*, the *Flaviviridae* family, and cause bovine viral diarrhea-mucosal disease (BVD-MD)^1^. BVD-MD is an acute, febrile, contact infectious disease, clinically manifests as acute diarrhea, respiratory disease, immunosuppression, and reproductive disorders^2^. BVDV has a wide host range, its infection has been detected not only in domesticated ruminants, but also in wild ruminants and wild boars^3^. BVDV is one of the major pathogens leading to the decline in cattle production performance. It is a serious threat to the development of animal husbandry in our country and even globally, resulting in severe economic losses. Bovine herpesvirus 1 (BoHV-1) is a member of the *Herpesviridae* family and genus *Varicellovirus*^4^. BoHV-1 is responsible for causing different syndromes, such as infectious bovine rhinotracheitis (IBR) and infectious pustular vulvo-vaginitis (IPV) in cows and balanoposthitis (IBP) in bulls^5^. BoHV-1 has been widely prevalent throughout the world with varying rates of positivity since it was first reported in 1953 in dairy cattle feedlots in Los Angeles, California^6^. To date, some countries have been able to eradicate IBR, such as Scandinavian countries (Denmark, Finland, Norway, and Sweden), as well as Austria, Switzerland, and some Italian regions^7^. Some studies have shown that BVDV and BoHV-1 usually coexist in diseased cattle. When cattle are infected with one virus, the immune system becomes weak, making it easier for other pathogens to enter the body, which is the main reason why these diseases are so difficult to eradicate^8^.

So far, detection methods for the diagnosis of these two viruses include virus isolation^9^, PCR techniques^10^, serological neutralization tests^11^, and enzyme-linked immunosorbent assays^12^. Virus isolation relies on live viruses to determine positive results, is expensive and time consuming^13^. The molecular biology assays detecting viral genomic DNA are either time-consuming or expensive or require sophisticated laboratory setup and skilled personnel^14^. The serologic methods depend on high quality antisera which are severely affected by improper sampling and autolysis, and are likewise more expensive^15^. These methods cannot accomplish rapid detection of clinical samples. Therefore, there is an urgent need to establish a rapid and accurate diagnostic method to provide a new generation of detection technology and means for the detection and control of bovine diseases.

Recombinase polymerase amplification (RPA) is a nucleic acid level detection technique different from PCR, which mainly involves three enzymes, namely, binding single-stranded nucleic acid recombinase (T32 UvsX), single-stranded DNA-binding protein (SSB), and strand-replacing DNA polymerase (Bsu polymerase)^16^. The RPA assay requires low temperature, short reaction time, simple operation, no special equipment and high stability^17^. Since its initial development, the RPA assay has been widely used for the detection of human, animal and plant pathogens such as Mycobacterium tuberculosis^18^, feline herpesviruses^19^, African swine fever virus^20^, and Yersinia coli^21^. The main commonly nucleic acid end-product assays are agarose gel electrophoresis(AGE)^16^, real-time fluorescence^22^, chemical color development^23^, electrochemistry^24^ and lateral flow test strips (LFD)^25^. Some studies have combined RPA with LFD for the detection of BoHV-1, but the LDF-RPA assay is relatively costly and not suitable for resource-limited areas^26^. Isothermal-based diagnostic techniques are single detection systems because they cannot detect multiple viruses at the same time and increase the output time of results in case of mixed infections. Nevertheless, AGE is the most commonly used method for nucleic acid end-product assay due to its lower cost in summer of equivalent conditions and simultaneous detection of multiple pathogens^27^. Multiple detection systems have been developed to detect multiple targets by detecting them in a single-tube reaction. Studies have reported the development of dual RPAs using animals and humans to detect pathogens including bacterial tick-borne diseases^28^,Chlamydia trachomatis (CT) and Neisseria gonorrhea (NG) in humans^29^, and major bacterial pathogens of bovine respiratory diseases^30^. Although RPA assay of BoHV-1 has been studied, BVDV and BoHV-1 usually present as mixed infections^31^. Therefore, for the first time, we developed a simple and efficient RPA assay for the simultaneous detection of BVDV and BoHV-1 for the first time, which provides technical support for the diagnosis of bovine diseases and epidemiologic risk assessment.

## Results

### Construction and characterization of the pcDNA 3.1-BVDV and pcDNA 3.1- BoHV-1 recombinant plasmids

**Figure 1:**
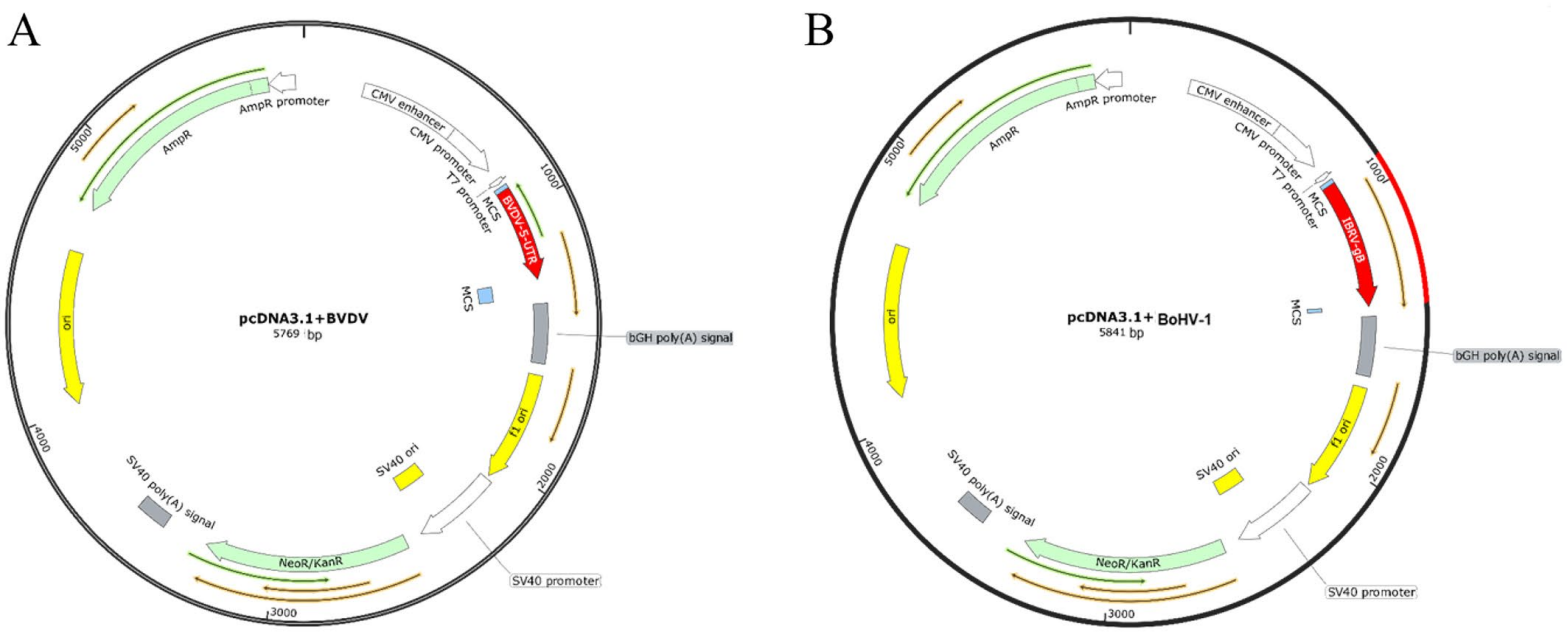
The map of pcDNA 3.1-BVDV and pcDNA 3.1-BoHV-1 recombinant plasmids. (**A**) pcDNA 3.1-BVDV recombinant plasmid mapping. (**B**) pcDNA 3.1- BoHV-1 recombinant plasmid mapping.

**Figure 2:**
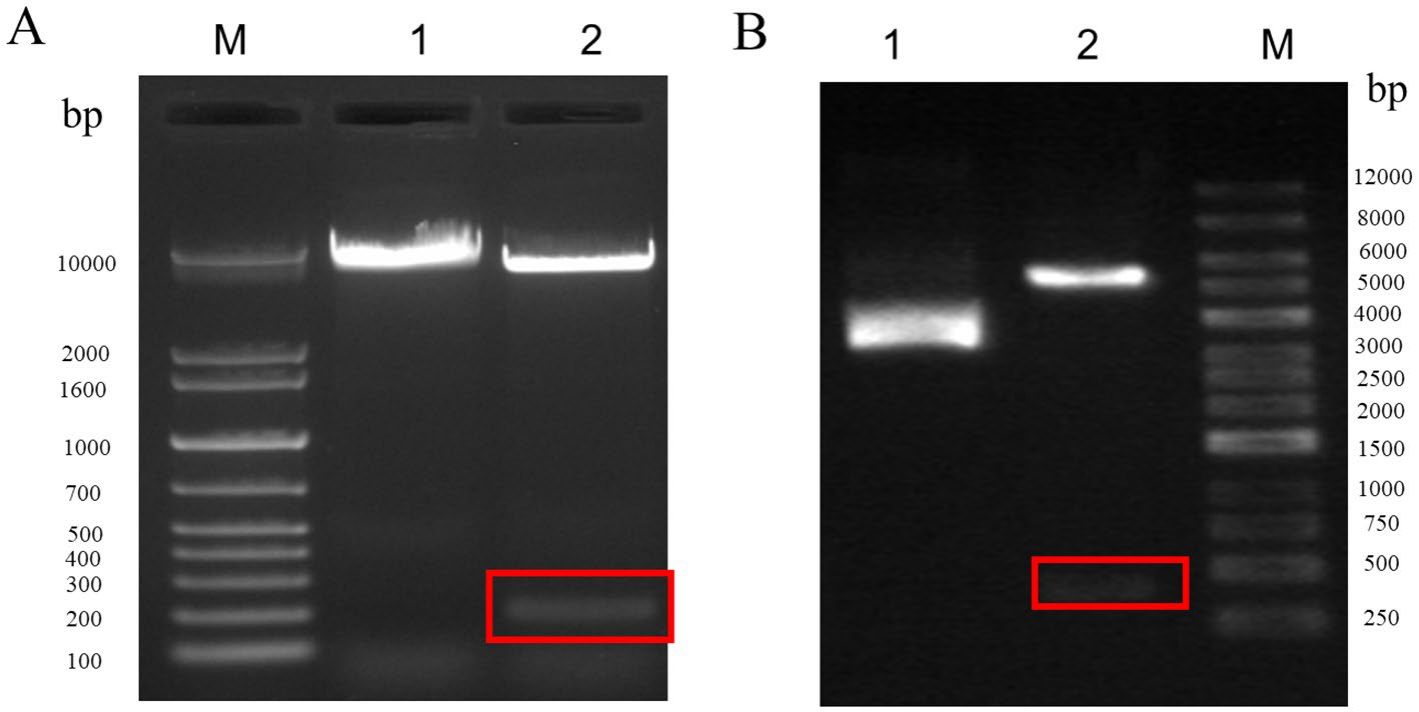
Recombinant plasmid digest identification. (**A**) pcDNA 3.1-BVDV digest identification, Lane M. 1 kb plus DNA Marker; Lane 1. *Hind III* single digest; Lane 2. *Hind III-Xhol* double digest. (**B**) pcDNA 3.1-BoHV-1 digest identification Lane M. 1 kb DNA marker; Lane 1. *Hind III* single digest; Lane 2. *Hind III-Bam HI* double digest. Insert is highlighted by a red box.

The BVDV *5'UTR* was constructed into the pcDNA 3.1 by *Bam HI* and *Hind III* restriction sites to obtain the pcDNA 3.1-BVDV recombinant plasmid. The BoHV-1 *gB* gene sequence was constructed into pcDNA 3.1 plasmid by *Hind III* and *Xhol* restriction sites to obtain pcDNA 3.1-BoHV-1 recombinant plasmid. The map of the plasmids is shown in Fig. 1. The results obtained from AGE (1%) of pcDNA 3.1-BVDV and pcDNA 3.1-BoHV-1 recombinant plasmids after digestion are shown in Fig. 2. The positions of the target bands were as expected. The pcDNA 3.1-BVDV recombinant plasmid showed a 365 bp band after double digestion, and the pcDNA 3.1- BoHV-1 recombinant plasmid showed a 493 bp band after double digestion. The sequencing results also matched the sequence of the target gene.

### Establishment and optimization of reaction conditions for BVDV and BoHV-1 dual RPA assay

**Figure 3:**
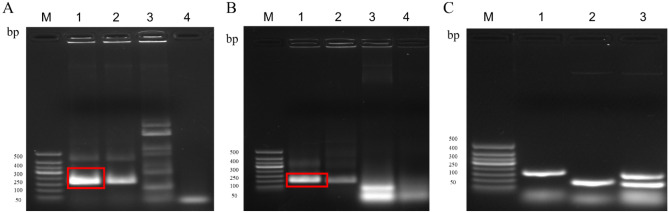
Primer screening for the dual RPA assay. (**A**) Primer screening for BVDV-RPA assay, Lane M. 50 bp DNA Marker; Lane 1. *5'UTR*-1 primers; Lane 2. *5'UT*R-2 primers; Lane 3. *5'UTR*-3 primers; Lane 4. Negative control. (**B**) Primer screening for BoHV-1 RPA assay, Lane M. 50 bp DNA Marker; Lane 1. *gB*-1 primers; Lane 2. *gB*-2 primers; Lane 3. *gB*-3 primers; Lane 4. Negative control. (**C**) BVDV and BoHV-1 dual RPA primer screen, Lane M. 50 bp DNA Marker; Lane 1. *5'UTR*-1 primers; Lane 2. *gB*-2 primers; Lane 3. *5'UTR*-1 primers and *gB*-1 primers premixes.

Candidate primers for BVDV, BoHV-1 RPA assay was selected by Twist Amp^®^ Basic reaction and initially analyzed on 2% agarose gel. The BVDV-RPA agarose gel results indicated that primer sets *5'UTR*-1F/R and *5'UTR*-2F/R produced specific amplification efficiency for RPA assay, both amplifying a 208 bp fragment. The primer set *5'UTR*-3F/R amplified a 206 bp fragment, but with more non-specific bands and primer dimerization (Fig. 3A). In addition, the *5'UTR*-1F/R primer set amplified the brightes and better amplification of the target band under the same conditions. Therefore, it was subsequently used in the BVDV-RPA assay. The agarose gel results of BoHV-1 RPA assay presented in Fig. 3B. Primer groups *gB*-1F/R and *gB*-2F/R produced specific amplification efficiency in the RPA assay, and the expected sizes of the products was 141 or 135 bp. Primer set gB-3F/R did not amplify the target band (139 bp) and showed a very distinct primer dimer that failed to meet the experimental requirements. Under the same conditions, *gB*-1F/R amplified the brightest and better amplification effect. Therefore, the *gB*-1F/R primer set was selected for subsequent use in the BoHV-1 RPA assay. Meanwhile, to verify the availability of the designed primers in BVDV and BoHV-1 dual RPA assay, the pcDNA 3.1-BVDV, pcDNA 3.1- BoHV-1 recombinant plasmid premixes were amplified by *5'UTR*-1F/R, *gB*-1F/R, respectively. As a result, the expected *5'UTR* (208 bp) and *gB* (141 bp) gene amplified fragments were successfully observed after using dual gene amplification primers (Fig. 3C).

**Figure 4:**
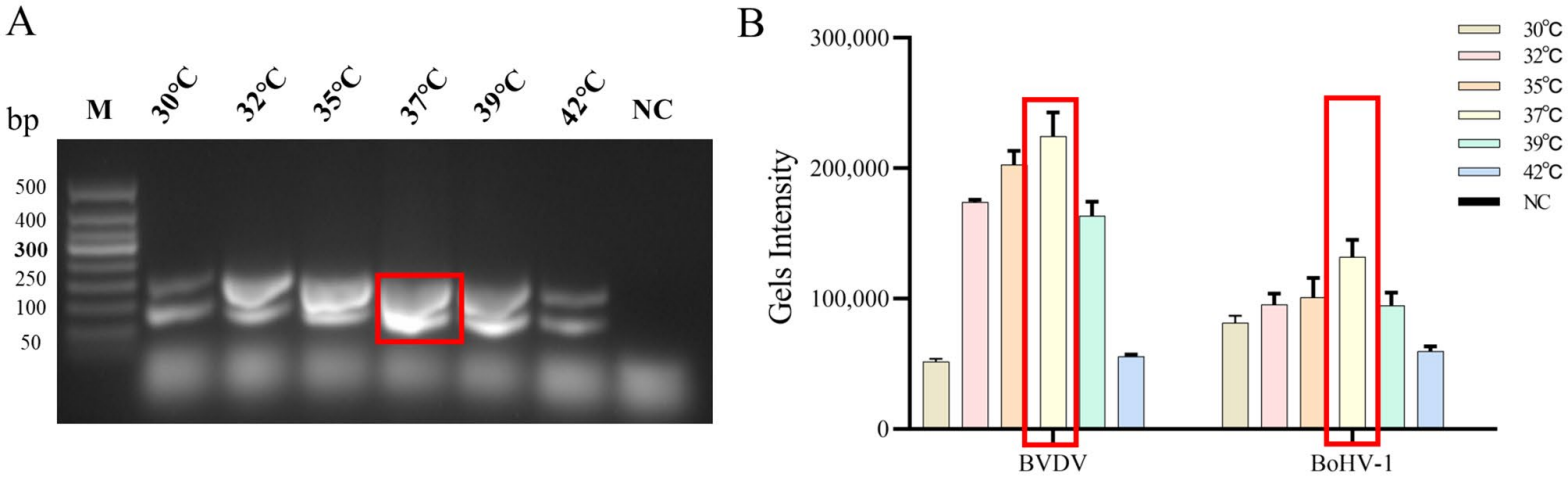
Reaction optimization of double RPA temperature conditions. (**A**) Double RPA temperature optimized gel electrophoresis. Lane M.DNA 50bp marker; Lane NC. Negative control. (**B**) Dual RPA temperature optimized gel intensity quantification.

**Figure 5:**
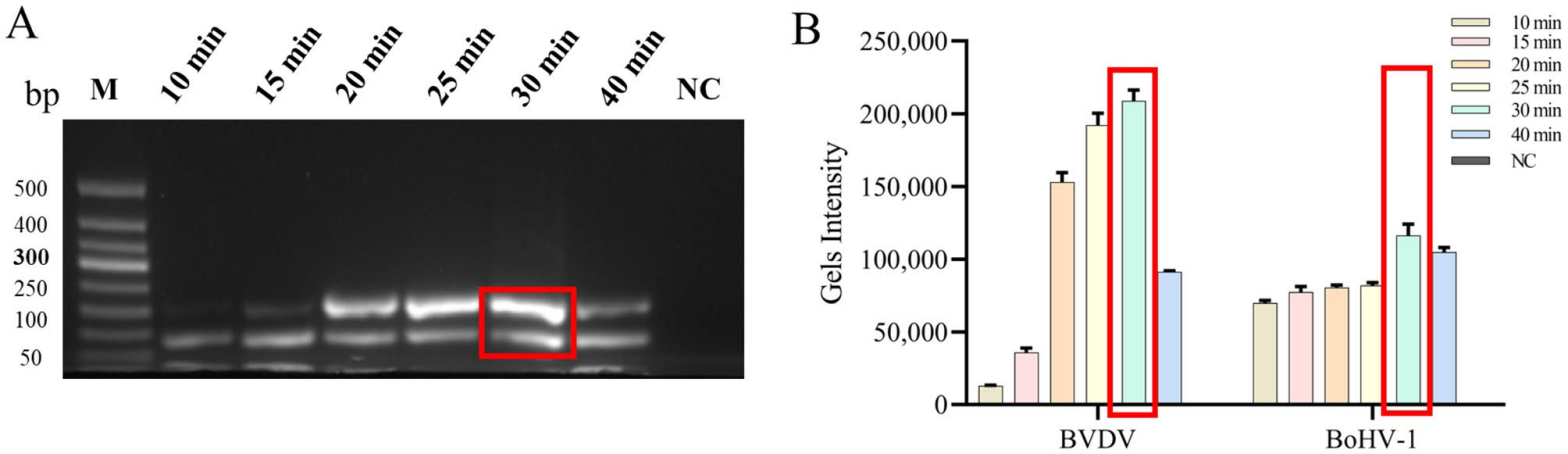
Reaction optimization of different time period of double RPA. (**A**) Dual RPA time-optimized gel electrophoresis. Lane M.DNA 50bp marker; Lane NC. Negative control. (**B**) Quantification of dual RPA time-optimized gel intensity.

The optimal reaction temperature for BVDV and BoHV-1 dual RPA assay were determined by testing different temperatures (30 °C, 32 °C, 35 °C, 37 °C, 39 °C, 42 °C), and reaction times (10 min, 15 min, 20 min, 25 min, 30 min, 40 min). As shown in Fig. 4, the target fragment gradually brightened with increasing reaction temperature, with the strongest color change at 37 °C. After that, the brightness of the target fragment decreased with increasing temperature, which may be caused by the inactivation of enzymes in the system due to high temperature, or high temperature makes the primers anneal less efficiently. Histogram analysis of the gel intensity by Image J analysis also showed that the BVDV and BoHV-1 RPA amplification was most efficient at 37 °C. Therefore, 37 °C was determined as the optimal reaction temperature. In addition, target fragment brightness was greatest at a reaction time of 30 min, and 30 min was the optimal reaction time (Fig. 5). Blank control tubes provided negative results under each condition. Finally, the optimal condition of 30 min reaction at 37 °C was selected for subsequent testing.

Different temperatures (30 °C, 32 °C, 35 °C, 37 °C, 39 °C, 42 °C) were set to determine the optimal reaction temperature for BVDV and BoHV-1 RPA assay. The negative control was incubated at 4 °C. As shown in Fig. 4, the temperature had a strong effect on the BVDV RPA assay, with the target bands becoming brighter as the temperature increased, and then darker after being brightest at 37 °C. The histogram of the gel intensity analyzed by Image J analysis also presented the same trend. Although the temperature has a small effect on the BoHV-1 RPA assay, the same conclusions can still be drawn as for the BVDV RPA assay. The brightness of the target fragment decreases with increasing temperature, which may be caused by the inactivation of enzymes in the system due to high temperature, or high temperature makes the primers anneal less efficiently. Therefore, 37 °C was determined as the optimal reaction temperature for BVDV and BoHV-1 RPA assays. The same method was applied with different reaction times (10 min, 15 min, 20 min, 25 min, 30 min, 40 min) to determine the optimal reaction time for the BVDV and BoHV-1 dual RPA assay. The negative control reaction time was 0 min. The BVDV RPA assay had almost no target bands and weak gel intensity after 10 min of amplification. Over time, the target fragment brightness was maximized at a reaction time of 30 min. The BoHV-1 RPA assay showed less variation over time, and quantitative analysis of gel strength revealed that gel strength was greatest at 30 min relative to other times (Fig. 5). Therefore, we chose the optimal condition of 30 min at 37 °C for the subsequent BVDV and BoHV-1 dual RPA assay.

### Sensitivity and specificity evaluation of the dual RPA assay

**Figure 6:**
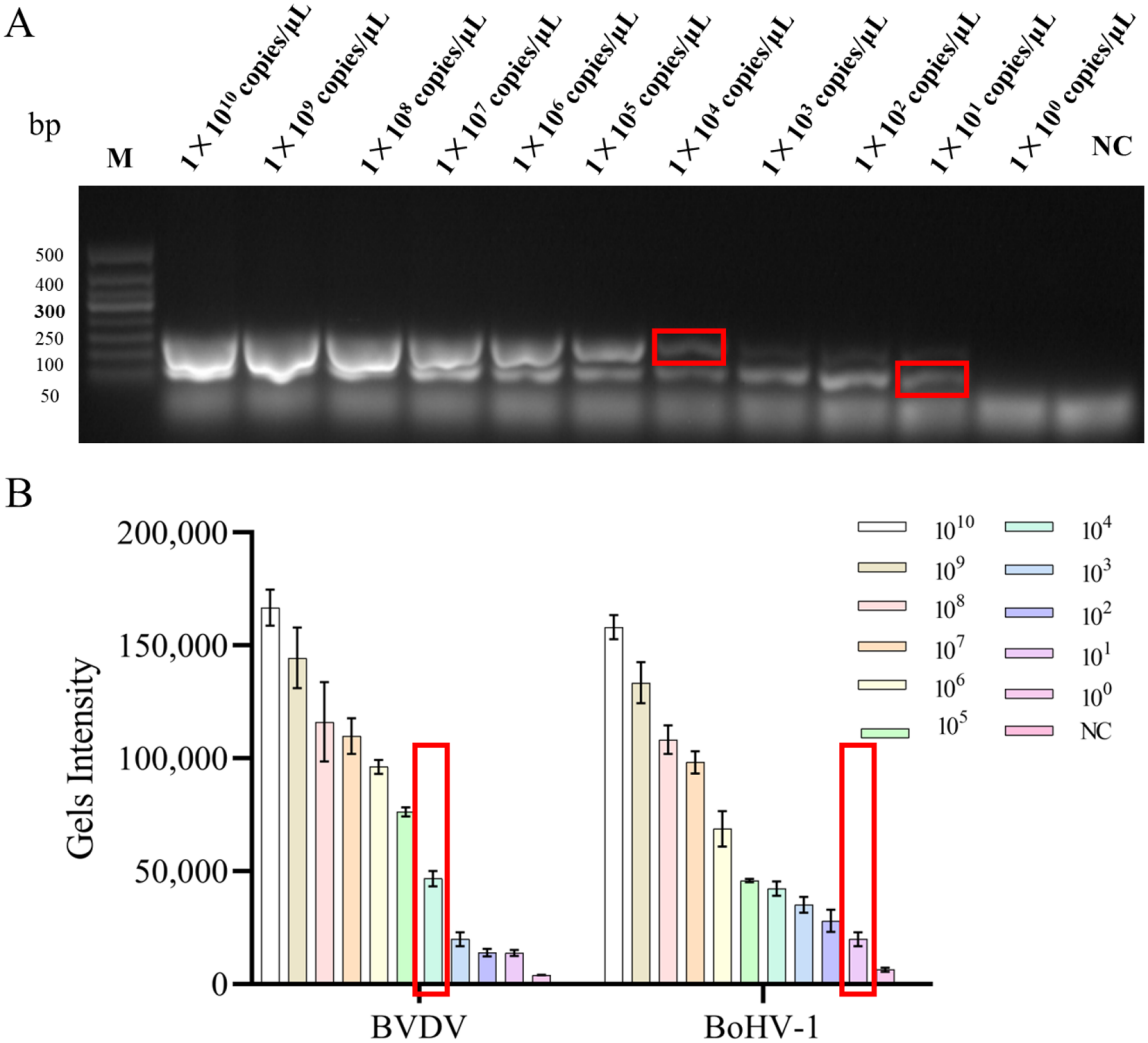
Sensitivity evaluation of dual RPA assay. (**A**) Gel electrophoresis diagram of double RPA sensitivity experiment. Lane M.DNA 50bp marker; Lane NC. Negative control. (**B**) Quantification of gel strength for double RPA sensitivity assay.

The quantified pcDNA 3.1-BVDV and pcDNA 3.1-BoHV-1 recombinant plasmids were serially diluted from 1 × 10^10^ copies/μL to 1 × 10^0^ copies/μL. The sensitivity test for BVDV and BoHV-1 dual RPA assay was performed by the electrophoretic detection technique. Nuclease-free water was used as a negative control. As shown in Fig. 6, the minimum detection limit for BVDV RPA-AGE was 1 × 10^1^ copies/μL, and that for BoHV-1 RPA-AGE was 1 × 10^4^ copies/μL.

**Figure 7:**
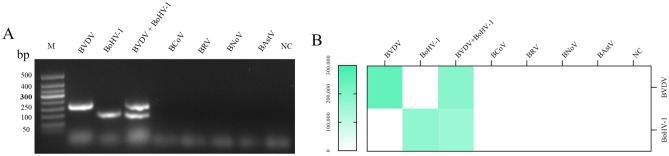
Specificity evaluation of the BoHV-1 RPA assay. (**A**) Specificity evaluation of the dual RPA assay. Lane M. DNA 50bp marker; Lane NC. Negative control. (**B**) Quantification of gel strength for the dual RPA specificity experiment.

The specificity of the dual RPA assay for BVDV and BoHV-1 was verified by RPA assays for six bovine respiratory and diarrhea-associated viruses (BVDV, BoHV-1, BCoV, BRV, BNoV, and BAstV). The results demonstrated that BVDV and BoHV-1 could be specifically detected using the established dual RPA. The distinctive bands of *5'UTR* or *gB* were clearly visible in the gel. No bands were amplified for other pathogens, such as BCoV, BRV, BNoV, BAstV, and nuclease-free water (Fig. 7). In summary, the established RPA assay can be used to specifically detect BVDV and BoHV-1 in single infection or co-infection.

### Dual RPA assay for BVDV and BoHV-1 in clinical samples

**Figure 8:**
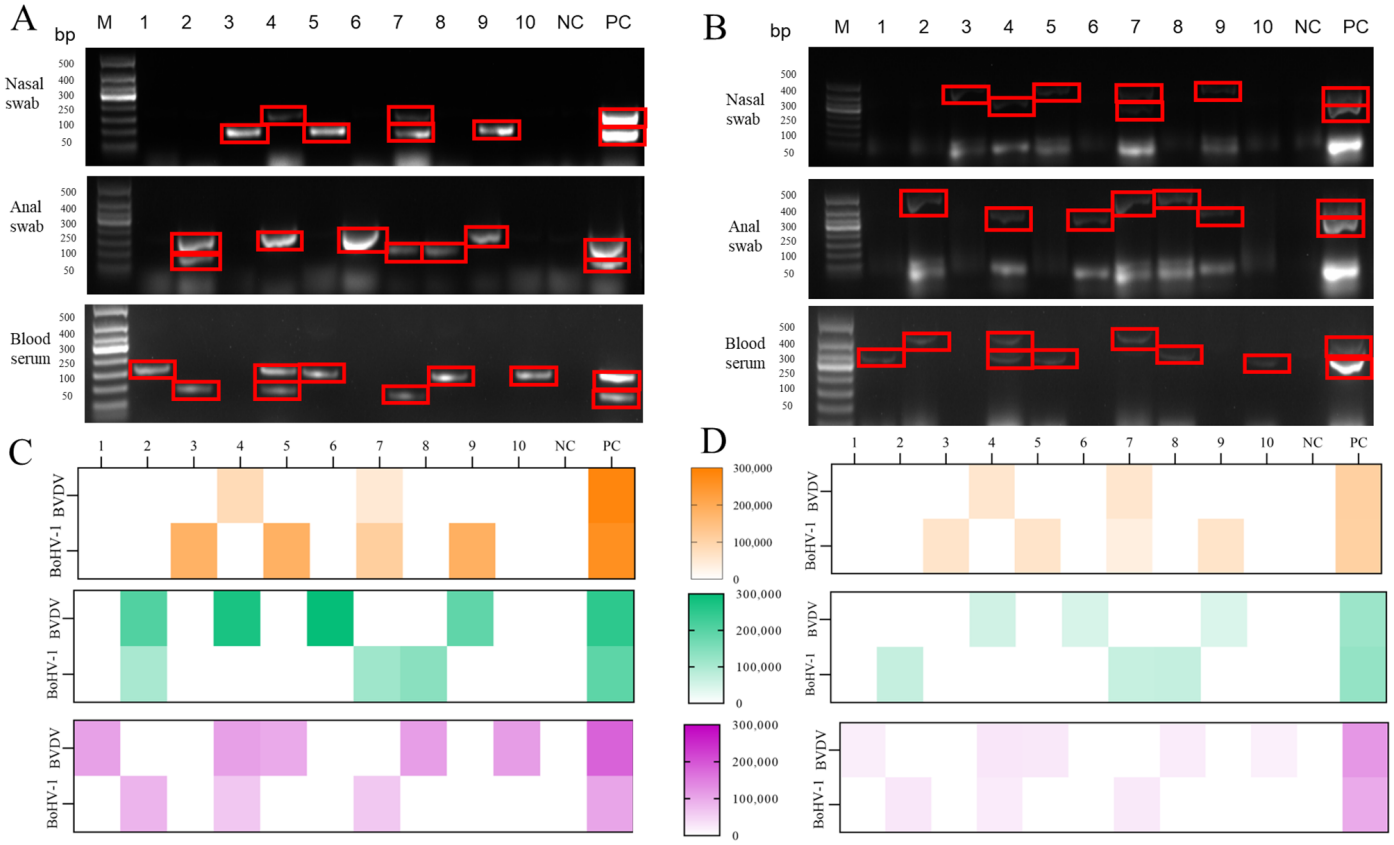
Dual RPA clinical sample detection. (**A**) Agarose gel plot of bovine clinical samples for dual RPA assay of BVDV and BoHV-1. The upper band is BVDV (208bp) and the lower band is BoHV-1 (141bp). (**B**) Agarose gel plot of BVDV and BoHV-1 PCR assays in bovine clinical samples. The upper band is BoHV-1 (481bp) and the lower band is BoHV-1 (354bp). (**C**) Gel strength quantification by BVDV and BoHV-1 dual RPA assays. (**D**) Gel strength quantification for BVDV and BoHV-1 PCR assays. Lane 1–10. Bovine clinical samples; Lane 11. Negative control; Lane 12. Positive control.

To evaluate the practical clinical application of the dual RPA assay for BVDV and BoHV-1, we performed the RPA assay on nasal swabs, anal swabs, and serum samples from cattle with significant respiratory and diarrheal disease. At the same time, the samples were tested using PCR to verify the RPA assay results. The results of RPA assay of clinical samples are shown in Fig. 8 and Table 1. The total positive rate of BVDV RPA assay of clinical samples was 36.67% (11/30). The positive rates of RPA in bovine nasal swabs, anal swabs and blood serum samples were 20%, 40% and 50%, and the positive rates of PCR were 20%, 30% and 50%. The positive rates of RPA and PCR assay in BoHV-1 clinical samples were the same as those of PCR, which were 33.33% (10/30). The RPA positivity of bovine nasal swabs, anal swabs and blood serum samples were 40%, 30% and 30%. Quantitative analysis of agarose gels revealed that the gel strength of the RPA assay was generally higher than that of PCR assay. Therefore, the established dual RPA method can be used as a candidate assay for the detection of BVDV and BoHV-1 in clinical samples.

**Table 1:** Clinical detection for BVDV, BoHV-1 dual RPAs.

Sample type	BVDV-RPA (Positive/total)	BVDV-PCR (positive/total)	BoHV-1 RPA (positive/total)	BoHV-1 PCR (positive/total)
Nasal swab	2/10	2/10	4/10	4/10
Anal swab	4/10	3/10	3/10	3/10
Blood serum	5/10	5/10	3/10	3/10
Total	11/30	10/30	10/30	10/30

## Discussion

BVDV and BoHV-1 are important pathogens in cattle with a global distribution that can cause significant economic losses^32^. BVDV isolates are further stratified into 3 species, BVDV-1 (1a–1 w, 23), BVDV-2 (2a–2d), and HoBi-like viruses (a–d)^1^. BVDV-1 has the highest occurrence in cattle population in China^33^. Therefore, rapid diagnosis of BVDV-1 epidemic strains has important significance in epidemiological studies. The *5*′*UTR* of BVDV is most often targeted for molecular diagnosis technology and genotyping since it is highly conserved in the pestivirus genome^34^. According to its antigenic and genomic characteristics, BoHV-1 is further subdivided into two distinct yet closely related subtypes: 1 (BoHV-1.1) and 2 (BoHV-1.2). Such subtypes may also be associated with distinct manifestations of the disease in cattle^35^. The gB gene is highly conserved among members of the Herpesviridae family and is frequently used as the target gene to detect BoHV-1^36^. During the development of the RPA assay, we chose the gB gene to design the primers.

Although vaccines against both viruses are available, such as the traditional modified live vaccine (MLV), inactivated vaccine (KV), DIVA (differentiate infected from vaccinated animals) vaccine and subunit vaccine^37^. However, the prevention and control of bovine diseases still require the establishment of rapid and reliable nucleic acid detection methods. RPA, an emerging isothermal nucleic acid amplification technique, has been applied to detect pathogens in a variety of samples, e.g., blood^38^, food^39^, and feces^40^. In our study, we established a dual RPA assay based on the BVDV *5'UTR* and BoHV-1 *gB* genes and validated it in bovine nasal swabs, anal swabs, and blood samples. In contrast, conventional PCR reactions require agarose gel electrophoresis and fluorescent quantitative PCR methods require thermal cyclers. In terms of reaction time, the RPA method (< 45 min) is less time-consuming than the PCR method^41^. In terms of experimental cost, compared to the cost of conventional PCR and real-time fluorescent quantitative PCR, although the RPA platform is more costly.

In addition to rapidity, specificity, and cost, we also tested the sensitivity of the RPA assay. The minimum detection threshold for BVDV-AGE was 1 × 10^1^ copies/μL, and that for BoHV-1-AGE was 1 × 10^4^ copies/μL. We established a dual PRA assay with a lower detection threshold for BVDV than RT-LAMP (4.67 × 10^0^) but higher than RT-PCR (4.67 × 10^3^), and a lower detection limit for BoHV-1 than qPCR (4.45 × 10^2^) but higher than LAMP (2 × 10^4^)^42,43^. The overall positivity rate for the BVDV RPA assay was higher in the clinical trials (36.67%) than the rate of the PCR assay (33.33%). One anal swab sample was positive by RPA assay and negative by PCR assay. Not only that, all other RPA gel electrophoresis intensities that tested positive were significantly higher than those of the PCR assay. It was found that the positive rate of serum samples was higher than that with anal swabs. The higher positivity rate of anal swabs than nasal swabs indicates that BVDV is present in the intestine as the major bovine diarrhea virus. The high positivity rate of blood serum samples could only be attributed to the presence of viral nucleic acid in the blood of cattle, not live virus. The positivity rate of bovine nasal swabs for BoHV-1 RPA and PCR were higher than that of anal swabs and serum samples, which also indicated that BoHV-1 mainly infected the respiratory tract of cattle.

However, the RPA assay still has some limitations. First, RPA assay may have false positives, and reaction tubes should be opened and closed carefully and gloves should be changed frequently. Second, the technique is expensive and not very popular. To reduce the cost, premixing of the RPA reaction system can be done in advance to reduce the total volume to 12.5 μL (1/4 of the original volume). In addition, the RPA assay also avoids nucleic acid extraction, thus allowing the user to easily perform multiple diagnostic assays^44^, which can be used to further reduce the cost of RPA assay. The success of multiplex RPA for simultaneous diagnosis of BVDV and BoHV-1 not only maintains the accuracy and sensitivity of the RPA assay, but also reduces the number of reagents and procedures required.

## Conclusion

In this study, an RPA assay was established for rapid, sensitive, and simultaneous detection of BVDV and BoHV-1 without the need for expensive laboratory equipment. The established RPA assay offers several advantages over other current detection techniques. The RPA method is time-saving and can be completed in less than 30 min. When the template plasmid was diluted to 1 × 10^1^ copies/μL using the RPA method, it demonstrated high sensitivity. The RPA technique does not require thermal cycling and is suitable for non-instrumented nucleic acid amplification platforms. In conclusion, this is a rapid and effective method for the simultaneous detection of BVDV and BoHV-1, especially for resource-limited laboratories. To the best of our knowledge, this study is the first report on simultaneous detection of BVDV and BoHV-1 using RPA.

## Methods

### The appropriate ethics declarations

The experimental protocol for the collection of bovine clinical samples was strictly in accordance with the Procedures and Guidelines for Animal Ethics in the People's Republic of China. The animal experimental protocol was approved by the Science and Technology Ethics Committee of Ningxia University (Code: NXU-2024-003).

All methods are reported in accordance with ARRIVE guidelines.

### Viruses and clinical samples

The BVDV *5'UTR* gene sequence information was based on the BVDV-1 strain (MA/101/05, GenBank ID: MW054940.1) and the BoHV-1 *gB* gene sequence information was based on the BoHV-1 strain (BRV/2018/SMU6352, GenBank ID: MK654723.1). BVDV, BoHV-1, Bovine coronavirus (BCoV/CH/NX-GY-1/2022, GenBank ID: OQ513841.1), bovine rotavirus (BRV/CH/NX-1/2022, GenBank ID: OQ513855.1), and bovine norovirus (BNoV/CH/NX-GY-5/2022, GenBank ID: OQ430676.1), and bovine astrovirus (BAstV/CH/NX- 1/2022) used for specificity evaluation in this study were kept by the laboratory.

The clinical samples were 30 samples of cattle with respiratory disease or diarrhea syndrome collected from different farms in the Ningxia region (July 2022), including 10 nasal swabs, 10 anal swabs, and 10 serum samples. The cattle sampled were approximately 24-week-old bulls of the Simmental breed and had no history of BVDV and BoHV-1 vaccination. All samples were stored at -80 °C before being used for further testing and analysis.

### Construction of standard plasmids

The BVDV *5'UTR* gene sequence and the BoHV-1 *gB* gene sequence were synthesized artificially due to the limitation of the pathogen source. The whole genome sequences of BVDV and BoHV-1 were collected in GenBank, and the sequences were compared and analyzed by Mega software. The intraspecies-conserved and interspecies-specific *5'UTR* (GenBank ID: MW054940.1) and *gB* gene (GenBank ID: MK654723.1) were selected as target genes for the RPA assay. The target genes were constructed into pcDNA-3.1 by *Hind III*, *Xhol*, and *Bam HI* enzymes (NEB, No. R0104/ R0146/ R0136), and transformed into *E. coli* recipient cells Top10, obtain pcDNA 3.1-BVDV, pcDNA 3.1-BoHV-1 recombinant plasmids.

Plasmid extraction was performed according to the Axygen Plasmid Extraction Kit (item no. AP-MN-P), and the concentration measured by Nanodrop ND-8000 spectrophotometer (Thermo Scientific, Dreieich, Germany). The DNA copy number was calculated according to the following formula: DNA copy number = (M × 6.02 × 10^23^ × 10^–9^)/ (n × 660), where M is the amount of DNA in nanograms, and n is length of the plasmid in bp. DNA standards were stored at −20 °C until further experiments.

### Primer design

The RPA primers were manually designed based on the conserved regions of the BVDV *5'UTR* gene, BoHV-1 *gB* gene, with reference to the instruction manual provided by the RPA reaction manufacturer Twist Dx (Cambridge, UK). Meanwhile, common PCR primers were designed for the constructed plasmid sequences (Table 2). All primers were synthesized by Sangon Biotech (Shanghai, China) and screened by observing their performance on 2% agarose gels.

**Table 2:** Primer information of the dual RPA assay for BVDV and BoHV-1.

Primer name	Primer sequence (5′–3′)	Length (bp)
5'UTR-1F	AGCTACTCGCCGGAGCGCTTCCAGCAGATC	208
5'UTR-1R	CTTGGGCACCCAGTCCCAGGCTACCGTCAC	
5'UTR-2F	ACTCGCCGGAGCGCTTCCAGCAGATCGAGG	208
5'UTR-2R	TGGGCACCCAGTCCCAGGCTACCGTCACGT	
5'UTR-3F	AGCTACTCGCCGGAGCGCTTCCAGCAGATC	206
5'UTR-3R	TGGGCACCCAGTCCCAGGCTACCGTCACGT	
gB-1F	AGCTACTCGCCGGAGCGCTTCCAGCAGATC	141
gB-1R	CTTGGGCACCCAGTCCCAGGCTACCGTCAC	
gB-2F	ACTCGCCGGAGCGCTTCCAGCAGATCGAGG	135
gB-2R	TGGGCACCCAGTCCCAGGCTACCGTCACGT	
gB-3F	AGCTACTCGCCGGAGCGCTTCCAGCAGATC	139
gB-3R	TGGGCACCCAGTCCCAGGCTACCGTCACGT	

### Establishment of a dual RPA assay for BVDV and BoHV-1

The RPA assay was performed using the extracted recombinant plasmids pcDNA 3.1-BVDV and pcDNA 3.1-BoHV-1 as templates according to the instructions of the Twist Amp^®^ Basic RPA kit (Twist Dx, item no. 10270-106). The following RPA reaction system was configured according to the recommendations of the kit: 2 μL of template, 11.2 μL of sterile deionized water, 29.5 μL of RPA reaction buffer, 2.4 μL each of the F and R primers at 10 pM, and 2.5 μL of magnesium acetate solution, which was mixed homogeneously and then fully solubilized in 4 mg of RPA basic lyophilisate. The RPA reaction was programmed to incubate at 39 °C for 20 min in a thermocycler (Bio Metra GmbH, 844-070-882). The amplification products were subjected to 2% agarose gel electrophoresis and the results were visualized and photographed in a UV gel imaging system.

The main parameters of the RPA assay were amplification temperature and reaction time. To optimize the BVDV, BoHV-1 dual RPA assay, experiments were performed at different reaction temperatures (30 °C, 32 °C, 35 °C, 37 °C, 39 °C, 42 °C) and reaction times (10 min, 15 min, 20 min, 25 min, 30 min, 40 min) according to the manufacturer's recommended protocol. The optimal reaction was determined based on the specific bands in the agarose gel. To quantify the gel imaging results, the target bands (grayscale images of line regions and rectangular selections) were analyzed using ImageJ software. Intensity data were analyzed with Graphpad Prism.

### Sensitivity, and specificity evaluation of BVDV and BoHV-1 dual RPA assay

The specificity of the dual RPA assays for BVDV and BoHV-1 was evaluated in bovine pathogens with similar clinical signs. BVDV, BoHV-1, BCoV, BRV, BNoV, and BAstV were detected according to established RPA assays. Nuclease-free water was used as a non-template control in the assay.

To investigate the sensitivity of the dual RPA assay for BVDV and BoHV-1, the quantified pcDNA 3.1-BVDV and pcDNA 3.1-BoHV-1 plasmids were diluted in 11 gradients, i.e., 1 × 10^10^ copies/μL to 1 × 10^0^ copies/μL. The negative control template was still nucleic acid-free water.

### Evaluation of the dual RPA assay using field samples

To effectively examine the effect of the dual RPA assay for BVDV and BoHV-1 established in this study on field samples, we tested 30 samples (10 nasal swabs, 10 anal swabs, and 10 sera) of bovine respiratory syndrome and diarrhea syndrome with obvious clinical symptoms in Ningxia (collected in July 2022) by RPA. The nuclease-free water was used as a negative control, the synthesized recombinant plasmids (pcDNA 3.1-BVDV and pcDNA 3.1-BoHV-1) were used as a positive control. In addition, a conventional PCR assay was performed on the same samples to compare the results with the RPA assay.

The BVDV conventional PCR primers are as follows. 5'UTR-F: TCTCGACCGGGGACATTATCT; 5'UTR-R: CATTCTGCAACGCGAAGGTG. The amplification size is 354 bp. The BoHV-1 conventional PCR primers are as follows. gB-F: GTACGACTCGTTCGCGCTCT; gB-R: CAAGTACGTCTCCAGGCTGCC. The amplification size is 481 bp.

Swab samples were generally resuspended in virus preservation solution. They are shaken for 10 min, centrifuged at 12,000 rpm for 10 min to remove solid precipitates, and filtered aseptically through a 0.22 μm membrane. Blood samples were centrifuged at 3000 rpm for 10 min after an overnight incubation at 4 °C, and the upper serum was aspirated for subsequent tests. The viral genomic DNA/RNA was extracted by the TianGen Viral Genomic DNA/RNA Extraction Kit (No. DP315) according to the manufacturer's instructions. The RNA was reverse transcribed into cDNA by the Prime Script™ II 1st Strand cDNA Synthesis Kit (No. 6210A) from TAKARA. The reverse transcribed cDNA and extracted DNA were subjected to RPA assay or PCR assay. The amplified products were detected by 2% agarose gel electrophoresis and quantitatively analyzed by ImageJ software.

### Statistical analysis

To visually determine the specificity, sensitivity, and evaluation of dual RPA in clinical samples, we quantified the gel imaging results and analyzed the target strips (grayscale images of line regions and rectangular selections) using ImageJ software. Intensity data were analyzed with Graphpad Prism. In addition, we cropped the gel imaging results and the original full gel map imaging is shown in the Supplementary Information.

### Supplementary Information

Supplementary Information.

## Data Availability

The dataset analyzed during the current study is available from the corresponding author on reasonable request. The nucleotide sequences under the relevant accession numbers (MW054940.1, MK654723.1) analyzed during the current study are available in the GenBank repository, https://www.ncbi.nlm.nih.gov/nucleotide/.
