# Supplementary material for: Simultaneous detection of bovine viral diarrhea virus (BVDV) and bovine herpesvirus 1 (BoHV-1) using recombinase polymerase amplification
Source: Sci Rep. 2024 May 3;14:10169. doi: 10.1038/s41598-024-56869-7 (PMC11068760; doi:10.1038/s41598-024-56869-7)
Supplement: Supplementary file 1 — Supplementary Information. [file 41598_2024_56869_MOESM1_ESM.pdf]

Original blots

The regions of the original blots used in main figures using red boxes

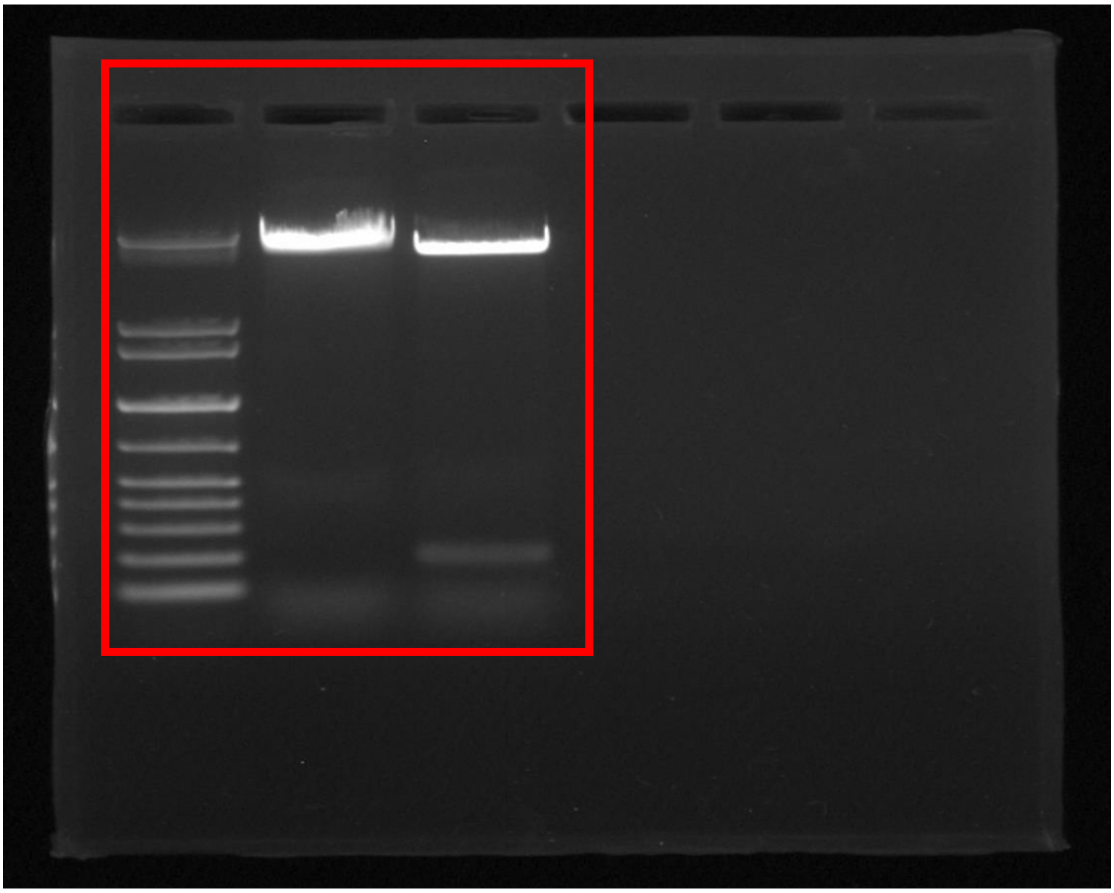

Figure 2A

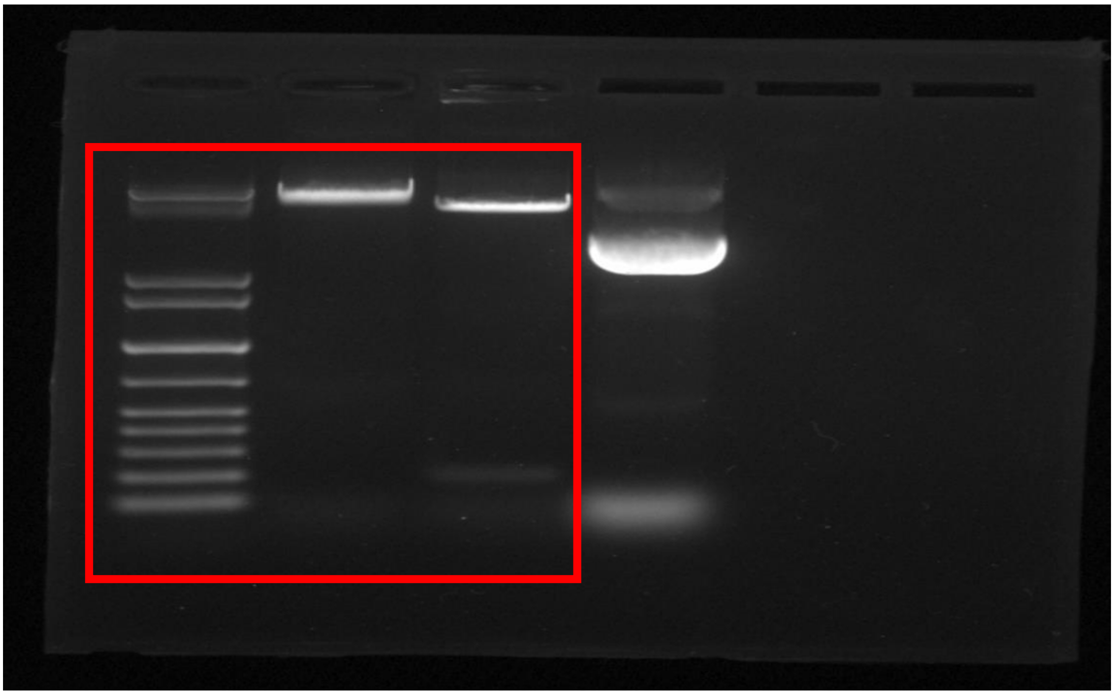

Figure 2B

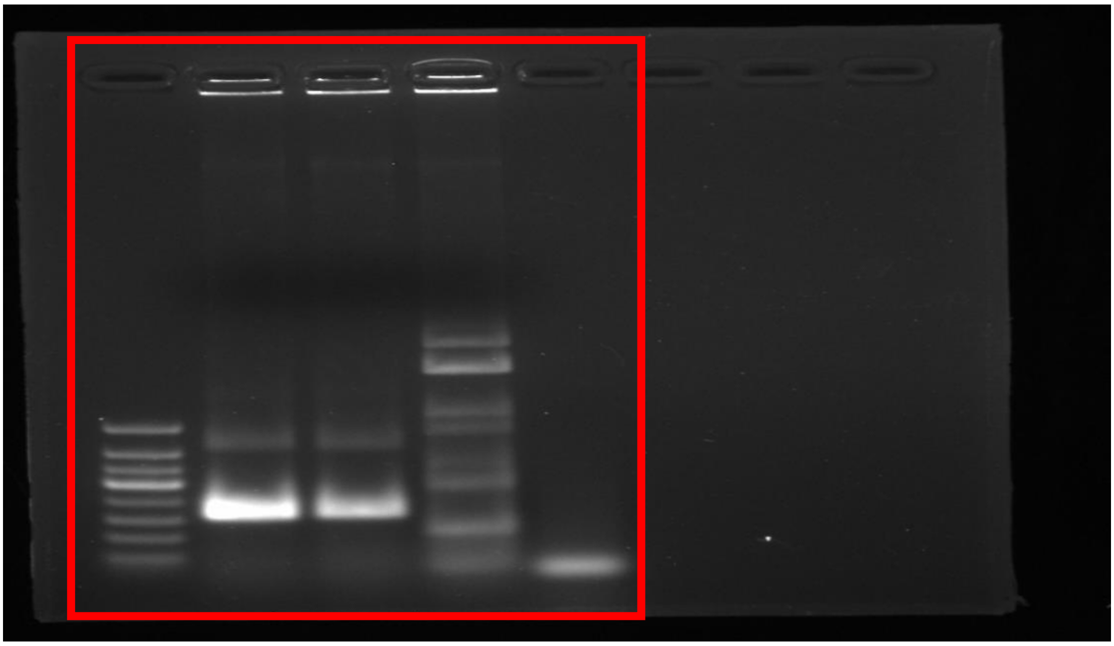

Figure 3A

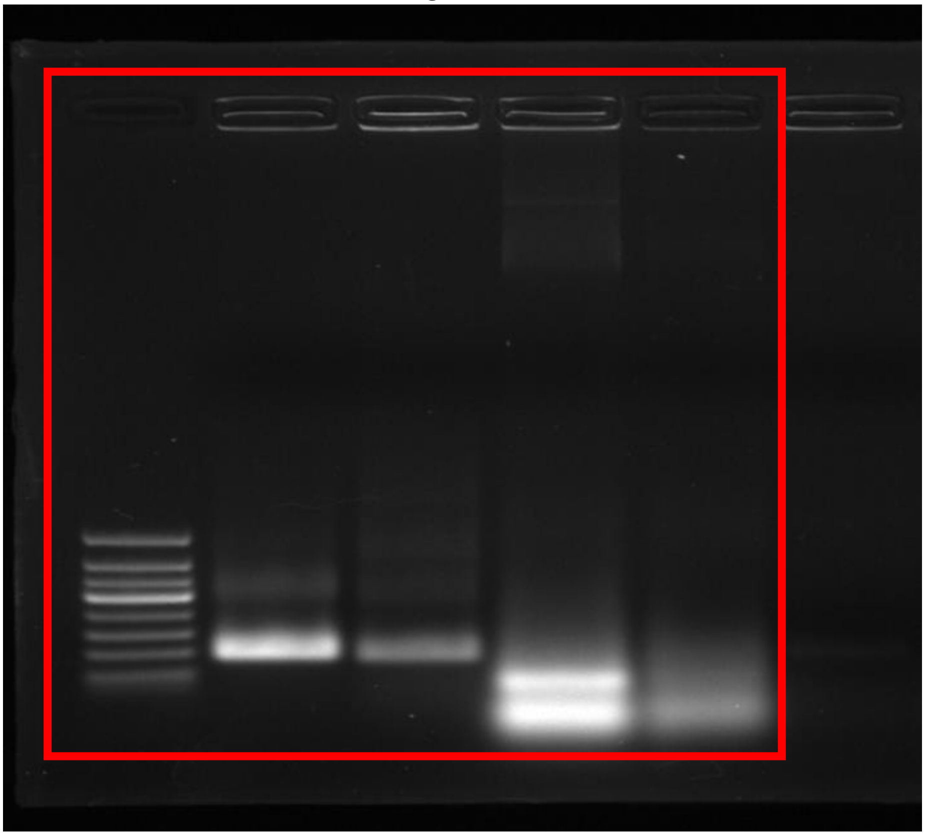

Figure 3B

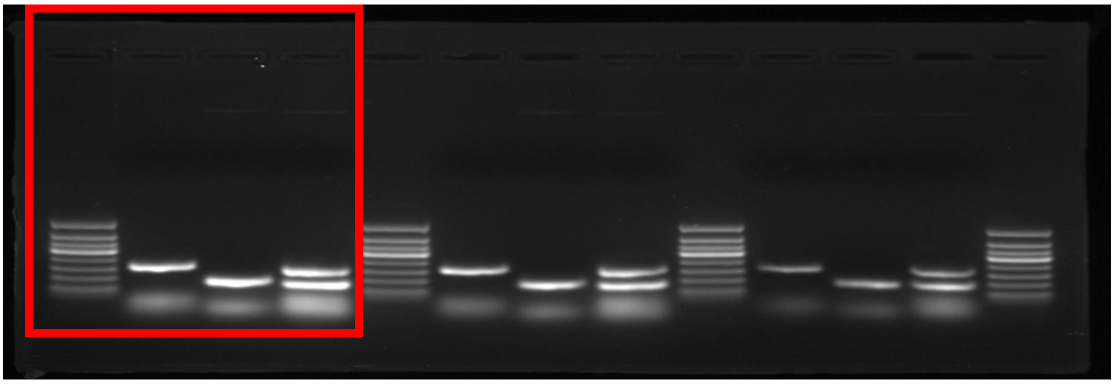

Figure 3C

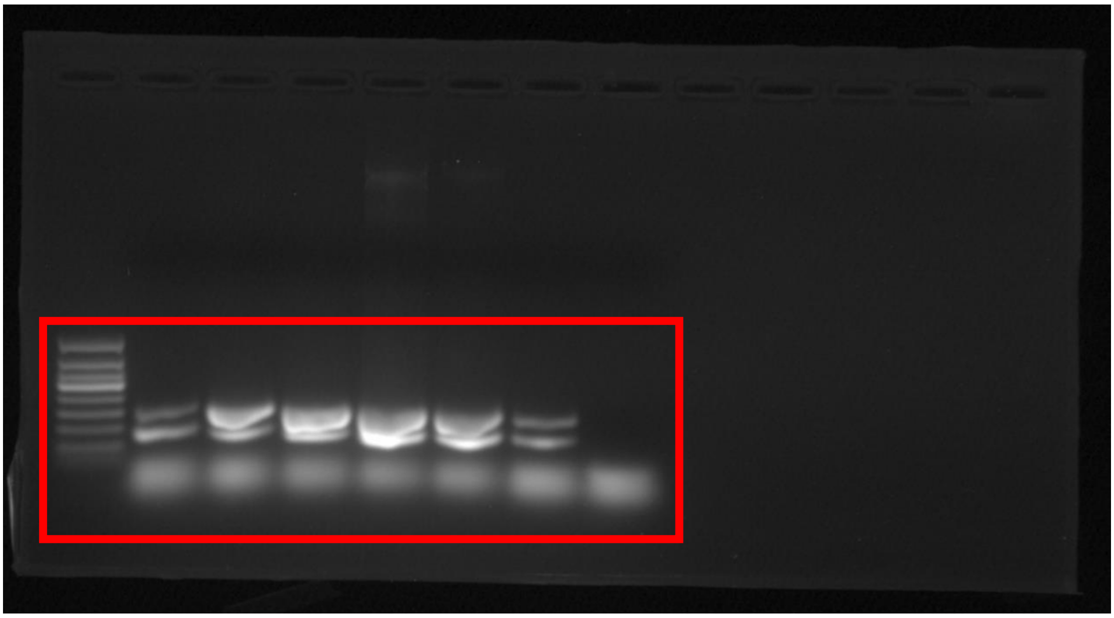

Figure 4A

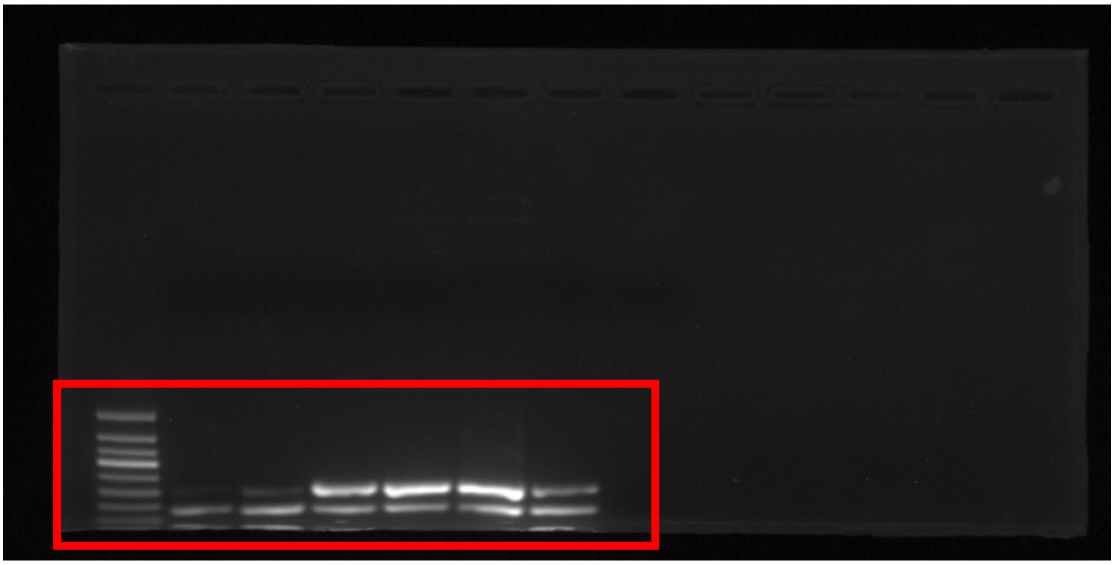

Figure 5A

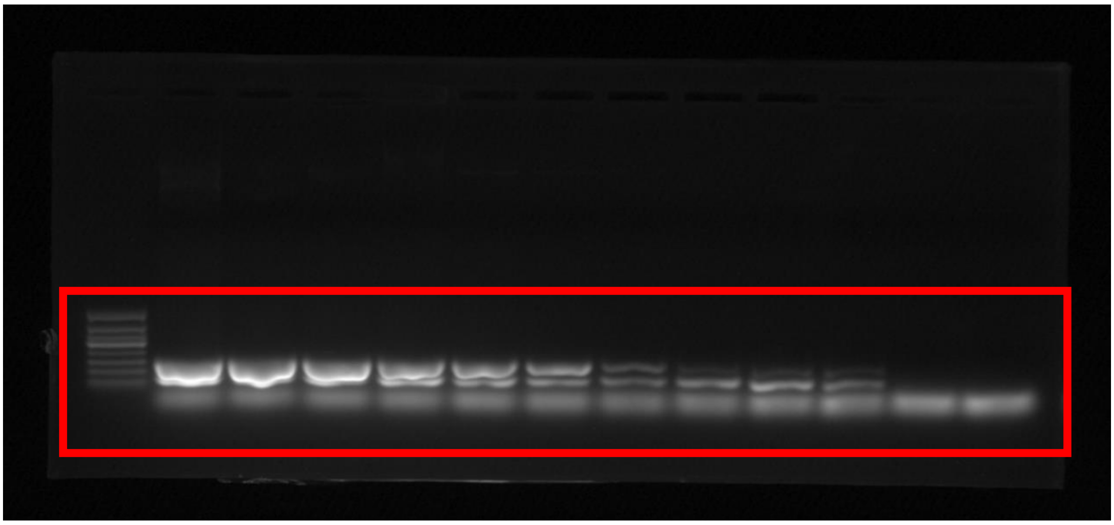

Figure 6A

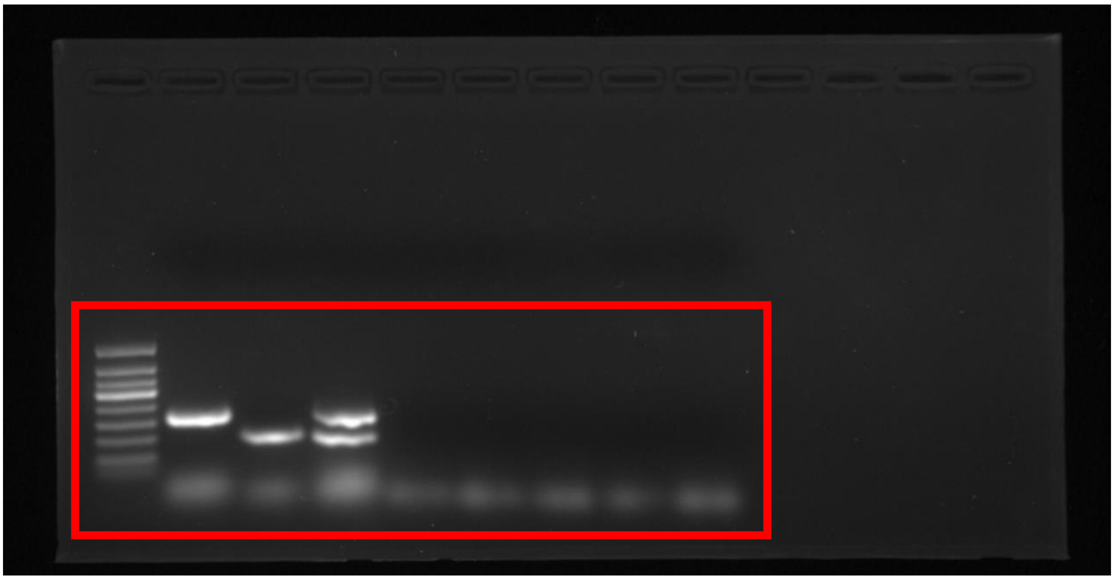

Figure 7A

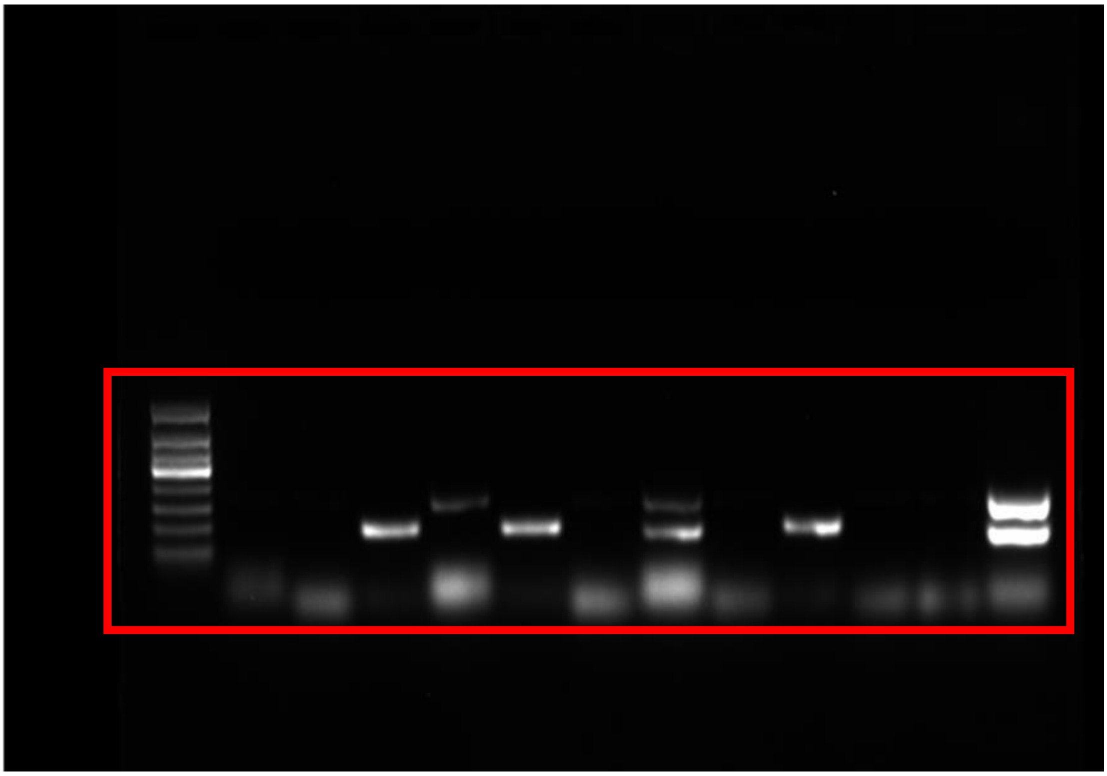

Figure 8A1

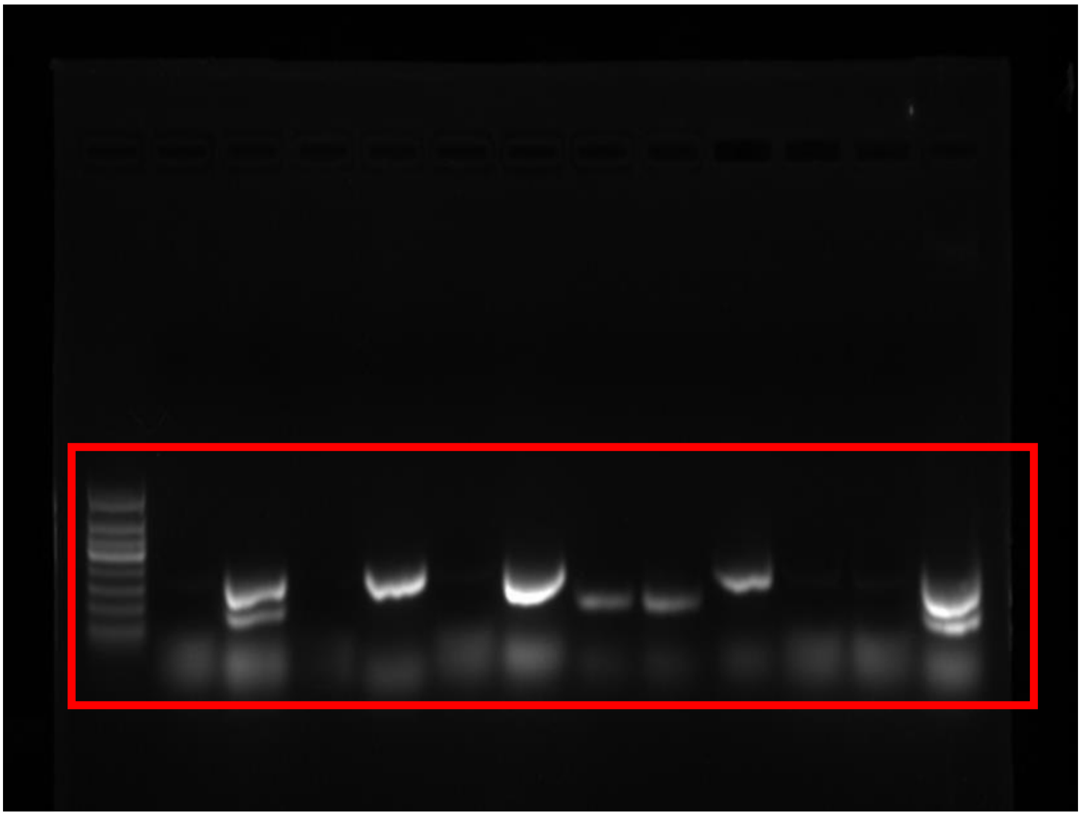

Figure 8A2

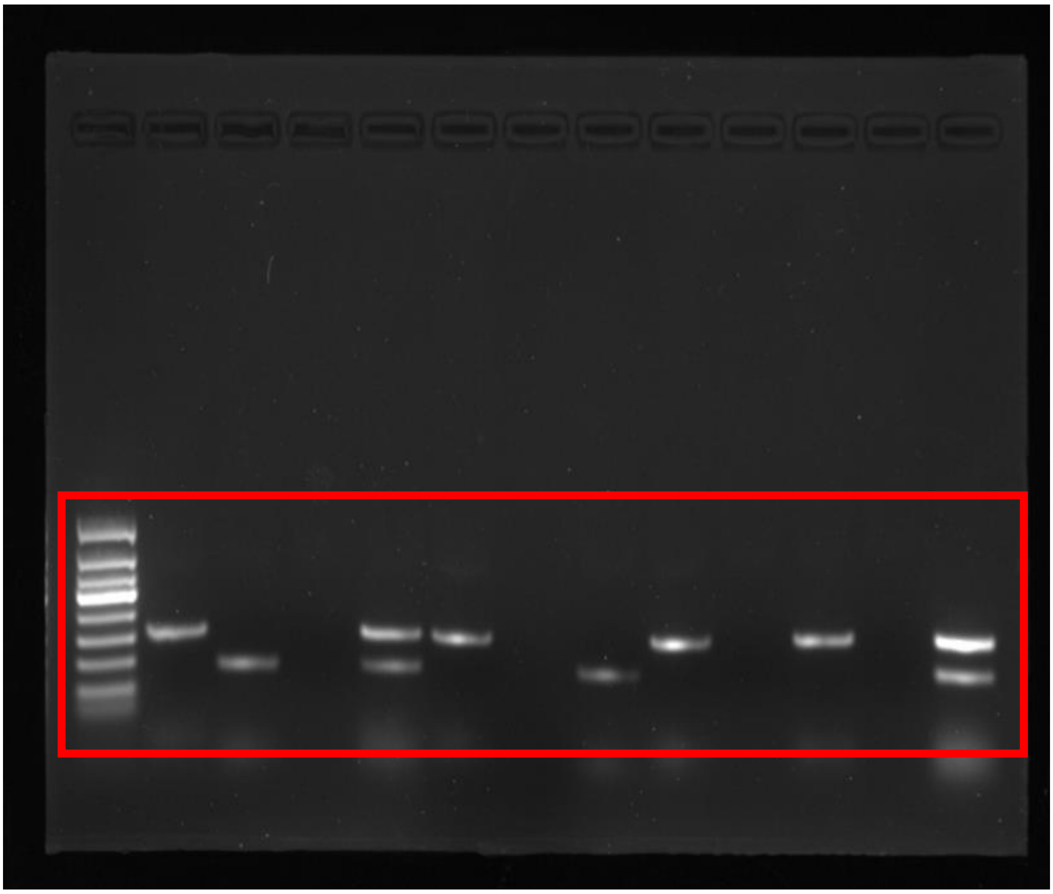

Figure 8A3

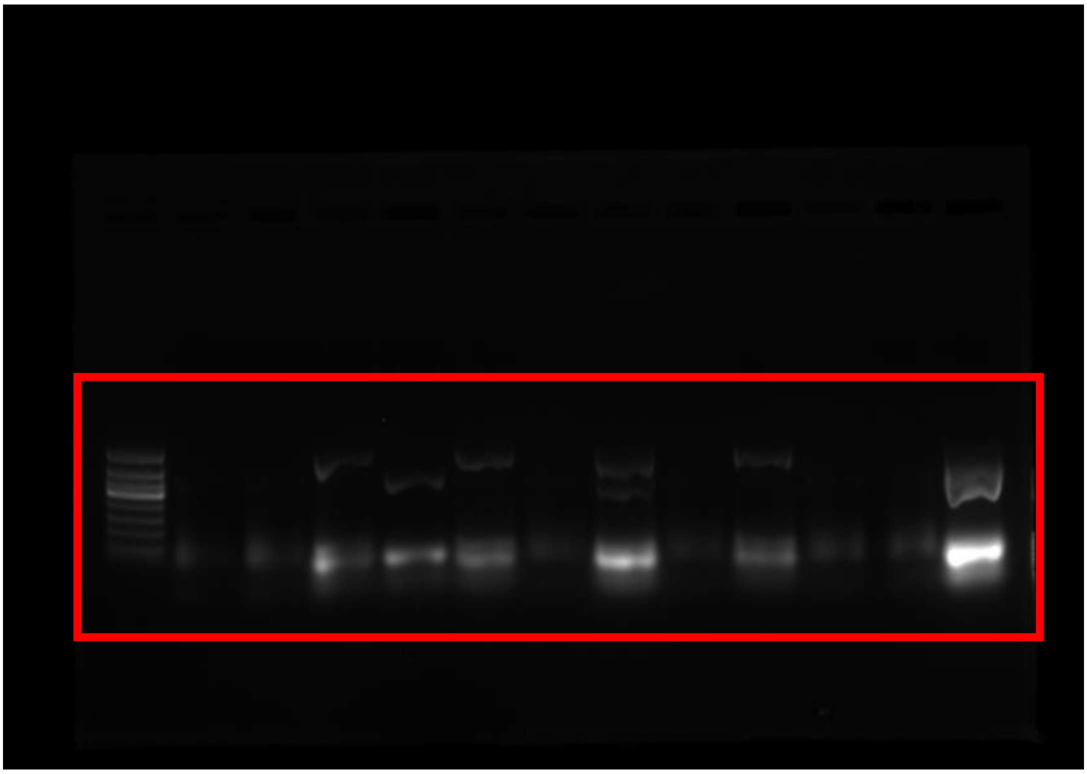

Figure 8B1

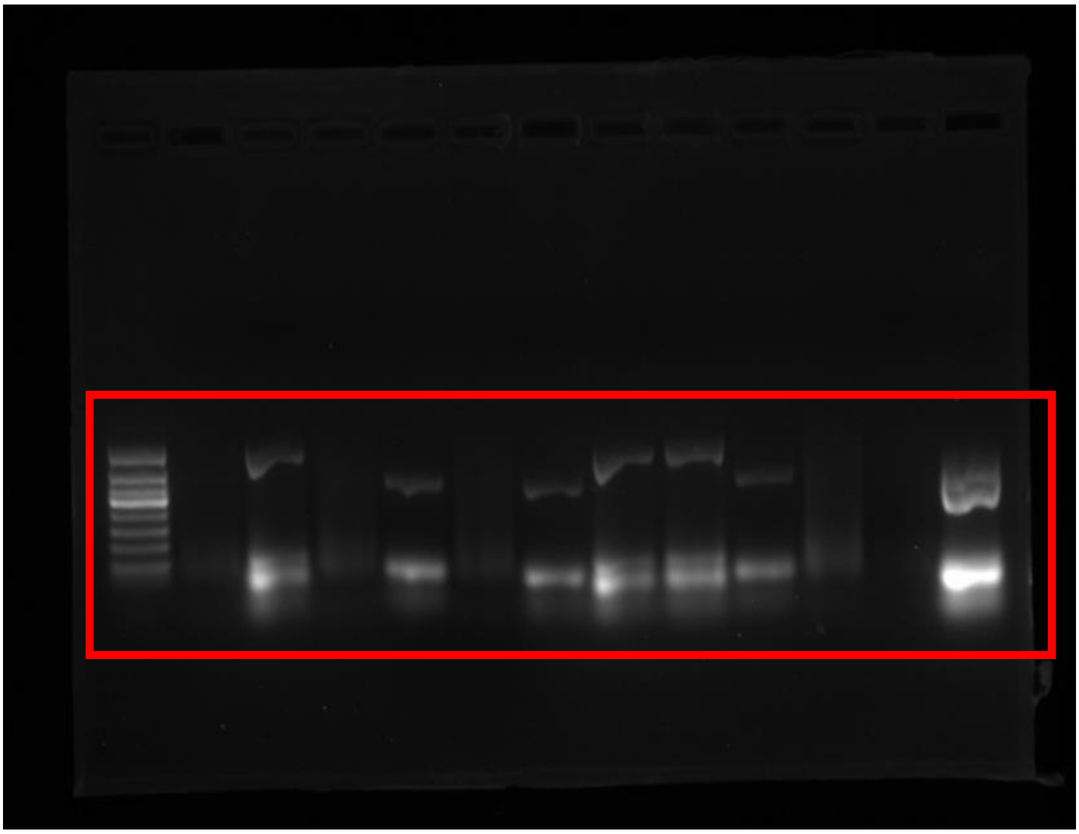

Figure 8B2

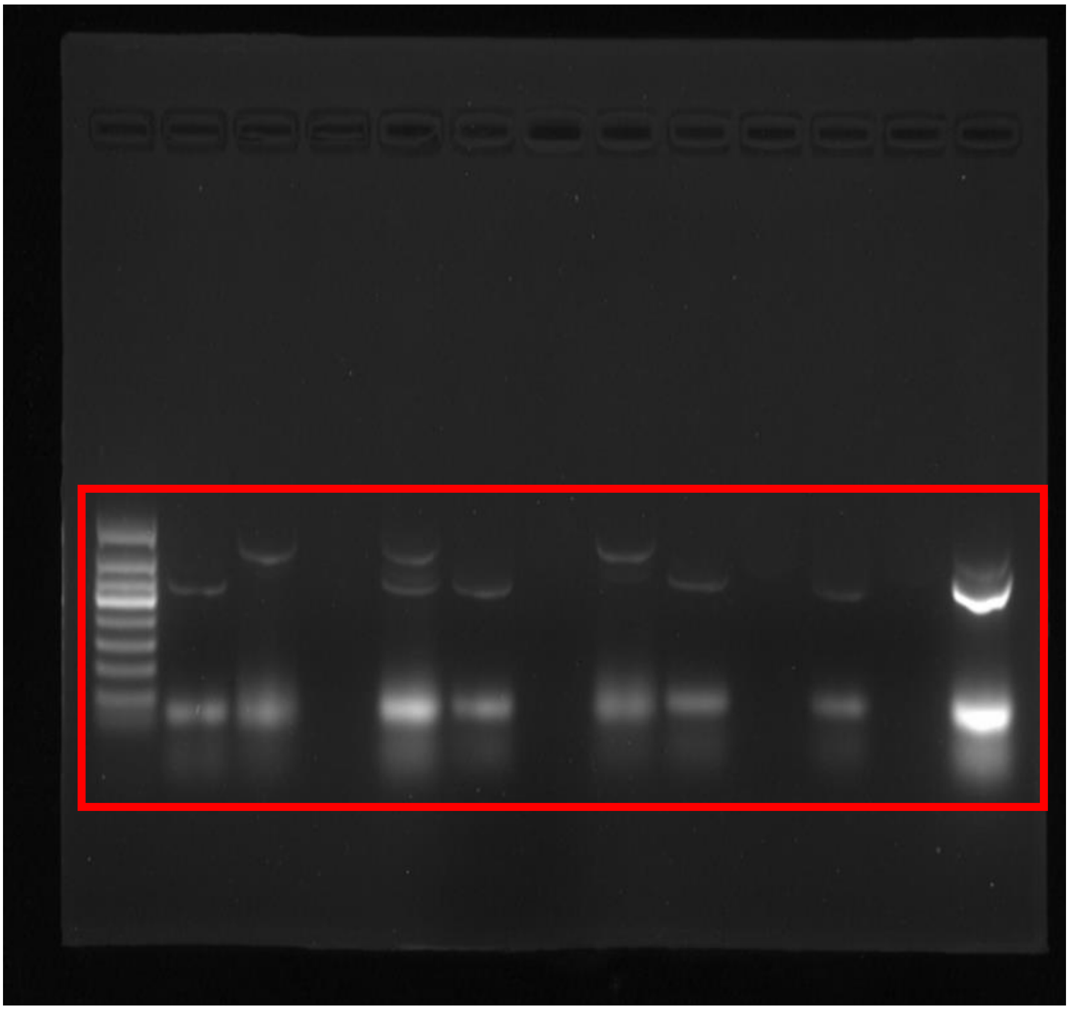

8B3
